# Differential expression of *PSMC4*, *SKP1*, and *HSPA8* in Parkinson’s disease: insights from a Mexican mestizo population

**DOI:** 10.3389/fnmol.2023.1298560

**Published:** 2023-12-05

**Authors:** Alma C. Salas-Leal, Sergio M. Salas-Pacheco, Erik I. Hernández-Cosaín, Lilia M. Vélez-Vélez, Elizabeth I. Antuna-Salcido, Francisco X. Castellanos-Juárez, Edna M. Méndez-Hernández, Osmel La Llave-León, Gerardo Quiñones-Canales, Oscar Arias-Carrión, Ada A. Sandoval-Carrillo, José M. Salas-Pacheco

**Affiliations:** ^1^Instituto de Investigación Científica, Universidad Juárez del Estado de Durango, Durango, México; ^2^Hospital General Santiago Ramón y Cajal-ISSSTE, Durango, México; ^3^Unidad de Trastornos del Movimiento y Sueño, Hospital General Dr. Manuel Gea González, Ciudad de México, México

**Keywords:** Parkinson’s disease, *UBE2K*, *PSMC4*, *SKP1*, *HSPA8*, protein degradation systems

## Abstract

Parkinson’s disease (PD) is a complex neurodegenerative condition characterized by alpha-synuclein aggregation and dysfunctional protein degradation pathways. This study investigates the differential gene expression of pivotal components (*UBE2K*, *PSMC4*, *SKP1*, and *HSPA8*) within these pathways in a Mexican-Mestizo PD population compared to healthy controls. We enrolled 87 PD patients and 87 controls, assessing their gene expression levels via RT-qPCR. Our results reveal a significant downregulation of *PSMC4*, *SKP1*, and *HSPA8* in the PD group (*p* = 0.033, *p* = 0.003, and *p* = 0.002, respectively). Logistic regression analyses establish a strong association between PD and reduced expression of *PSMC4*, *SKP1*, and *HSPA8* (OR = 0.640, 95% CI = 0.415–0.987; OR = 0.000, 95% CI = 0.000–0.075; OR = 0.550, 95% CI = 0.368–0.823, respectively). Conversely, *UBE2K* exhibited no significant association or expression difference between the groups. Furthermore, we develop a gene expression model based on *HSPA8*, *PSMC4*, and *SKP1*, demonstrating robust discrimination between healthy controls and PD patients. Notably, the model’s diagnostic efficacy is particularly pronounced in early-stage PD. In conclusion, our study provides compelling evidence linking decreased gene expression of *PSMC4*, *SKP1*, and *HSPA8* to PD in the Mexican-Mestizo population. Additionally, our gene expression model exhibits promise as a diagnostic tool, particularly for early-stage PD diagnosis.

## 1 Introduction

Parkinson’s disease (PD) stands as a multifaceted neurodegenerative disorder characterized by the progressive degeneration of dopaminergic neurons. Nevertheless, the intricate mechanisms underlying this condition remain shrouded in uncertainty. Emerging evidence has spotlighted dysregulation in the clearance and degradation of alpha-synuclein as a pivotal player in the pathogenesis of neuronal demise in PD (Mehra et al., 2019; Jankovic and Tan, 2020). At the crux of this degradation nexus lies the concerted interplay among chaperones, the ubiquitin-proteasome system (UPS), and autophagy-lysosomal pathways.

The UPS pathway orchestrates the disposal of damaged proteins through a meticulous tagging process involving three key enzymes: activating enzyme E1, conjugating enzyme E2, and ligase enzyme E3 (Chen and Dou, 2010). Subsequent to this tagging, the protein-ubiquitin conjugates are recognized and degraded by the 26S proteasome, a large proteolytic assembly resident within the cytosol and nucleus of eukaryotic cells. This proteasome is composed of a 20S core particle and a 19S regulatory particle (Jarome and Devulapalli, 2018).

Intriguingly, prior inquiries into the expression of genes associated with these degradation machineries in blood and brain tissues of individuals afflicted by PD, including *PSMC4* (an integral component of the 19S proteasomal particle), *SKP1* (an essential constituent of the SCF complex endowed with E3 ubiquitin ligase activity), *UBE2K* (an ubiquitin-conjugating enzyme E2K), and *HSPA8* (a 70kDa heat shock protein exerting chaperone functions), have yielded divergent results (Grunblatt et al., 2004; Mandel et al., 2005, 2012b; Molochnikov et al., 2012; Su et al., 2018). This divergence may stem from genetic disparities among diverse populations, imparting an element of complexity to our understanding of PD etiology.

Thus, the principal objective of our study is to scrutinize the expression profiles of these genes in peripheral blood samples sourced from the Mexican-mestizo population. By doing so, we aim to ascertain whether these gene expression patterns possess the potential to serve as a risk profile for PD.

## 2 Materials and methods

### 2.1 Study participants and ethical criteria

We collected samples from a cohort of 87 PD patients and 87 healthy, age-matched controls devoid of familial or personal histories of neurodegenerative diseases. PD diagnoses were confirmed by neurologists employing the United Kingdom Parkinson’s disease Society Brain Bank (UKPDSBB) diagnostic criteria. Participants were recruited from three prominent public hospitals located in central and northwest Mexico: Hospital General Dr. Manuel Gea Gonzalez in Mexico City, Hospital General 450, and Hospital General Santiago Ramón y Cajal in Durango City. This study secured the ethical approval of each participating hospital’s ethics committee.

All procedures adhered strictly to the ethical principles outlined in the 1964 Helsinki Declaration and its subsequent amendments. Informed written consent was obtained from all participants, with the additional requirement of a family member’s consent for each participant. Pertinent patient data, encompassing age, gender, presence of depression, cognitive status, age of disease onset, and Unified Parkinson’s disease Rating Scale (UPDRS) scores, were meticulously documented.

### 2.2 Blood collection, RNA extraction, cDNA synthesis, and RT-qPCR

Venous whole blood was collected from the subjects in Tempus Blood RNA tubes and reserved at −80 C until RNA extraction. We processed samples according to the manufacturer protocol to extract total RNA using MagMAX for Stabilized Blood Tubes RNA Isolation kit (Life Technologies, Norway). We then synthesized cDNA using a High-Capacity cDNA Reverse Transcription kit (Applied Biosystems, Carlsbad, CA, USA) according to the manufacturer’s instructions. Concentration and purity of RNA and cDNA were determined using NanoDrop 2000 spectrophotometer (Thermo Fisher Scientific Inc., Germering, Germany) and cDNA was stored at −20 C until further evaluation. Gene expression levels of *UBE2K, HSPA8, SKP1*, and *PSMC4* were measured through relative quantification (RQ) of 125 ng/mL of cDNA by Real-time quantitative PCR (RT-qPCR) using a QuantStudio 3 System (Applied Biosystems, Carlsbad, CA, USA) with TaqMan assays (*UBE2K* assay ID Hs00193507_m1, *HSPA8* assay ID Hs03044880_gH, *SKP1* assay ID Hs00429069_m1 and *PSMC4* assay ID Hs00197826_m1, Applied Biosystems). The expression was normalized to the *RPLP0* gene (*RPLP0* assay ID Hs00420895_gH, Applied Biosystems). The reaction conditions were: initial hold of 10 min at 95 C; 40 cycles of denaturation for 15 s at 95 C and annealing for 1 min at 60 C.

### 2.3 Statistical methods

Statistical analyses were executed using SPSS Statistics 20.0 software. Quantitative data are presented as mean ± standard deviation (SD). For normally distributed parameters, differences were assessed using a two-tailed t-test, while non-parametric tests were applied to analyze mRNA and protein levels. Logistic regression was employed to investigate the association between mRNA expression and PD, with significant P-values retaining relevance in constructing predictive classifier models. Receiver operating characteristic curve (ROC) data were derived from predictive probabilities generated via multivariate logistic regression, encompassing all PD patients and a subset of early-stage PD patients (those with less than 5 and 3 years of disease evolution). Correlations were evaluated via Spearman correlation with the two-tailed test of significance. *P*-value < 0.05 was considered statistically significant.

## 3 Results

### 3.1 Demographic and clinical characteristics

Table 1 presents a comprehensive overview of the demographic and clinical attributes of both the PD cases and control subjects. Comparative analysis of these variables between the two groups unveiled significant differences on the presence of depression (*p* = 0.040).

**TABLE 1:** Demographic and clinical characteristics of study participants.

	PD *n* = 87	Control *n* = 87	*p*
Age ± SD	70.4 ± 9.27	70.03 ± 9.19	0.793^+^
**Gender**			
Female	42 (48.3%)	42 (48.3%)	1^++^
Male	45 (51.7%)	45 (51.7%)	
Minimum Age	53	53	
Maximum Age	94	92	
Depression (HAM-D)	67 (77.2%)	53 (61.3%)	0.040^++^
Cognitive impairment (MMSE)	44 (51.2%)	45 (52.3%)	0.890^++^
Age of onset ± SD	64.9 ± 9.6		
PD Early stage (smaller or equal to 5 years of diagnosis)	52 (59.8%)		
PD Early stage (smaller or equal to 3 years of diagnosis)	31 (17.8%)		
UPDRS total score ± SD	68.6 ± 31.3		
UPDRS PART I ± SD	10.5 ± 6.9		
UPDRS PART II ± SD	14.9 ± 9.4		
UPDRS PART III ± SD	39.9 ± 19.7		
UPDRS PART IV ± SD	2.3 ± 4.8		

^+^Student t-test, ^++^chi-square test.

### 3.2 Gene expression profiles

**FIGURE 1:**
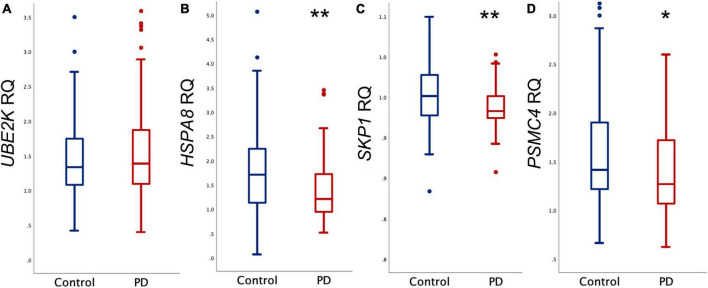
Relative quantification (RQ) of mRNA. Comparison between control and PD groups of mRNA levels of **(A)** *UBE2K*, **(B)** *HSPA8*, **(C)** *SKP1*, and **(D)** *PSMC4*. Outliers are denoted by dots. **p < 0.005, *p < 0.05.

Figure 1 provides a graphical representation of the observed gene expression levels in both the PD and control groups. Utilizing Relative Quantification (RQ) analysis, we identified a significant reduction in the expression levels of *HSPA8*, *SKP1*, and *PSMC4* among PD cases (*p* = 0.002, *p* = 0.003, and *p* = 0.033, respectively). To further elucidate their relevance to PD, logistic regression analysis was conducted, adjusting for sex and age (Table 2). The results unequivocally substantiated the associations of *HSPA8*, *SKP1*, and *PSMC4* with PD. However, *UBE2K* exhibited no statistically significant association with the disease.

**TABLE 2:** Association of gene expression levels with PD adjusted by sex and age.

	OR*	95% CI	*p*
*UBE2K*	0.943	0.711–1.250	0.681
*HSPA8*	0.550	0.368–0.823	0.004
*SKP1*	0.000	0.000–0.075	0.012
*PSMC4*	0.640	0.415–0.987	0.044

OR, Odds Ratio; CI, confidence interval. *Adjusted by sex and age.

### 3.3 Influence of depression on gene expression

We observed a notable disparity in the frequency of depression between the PD and control groups (Table 1), consequently, we conducted a gene expression association analysis considering while adjusting for sex, age, and depression. The findings revealed that, even after factoring in depression, the previously established associations for *HSPA8* and *SKP1* remained statistically significant (Table 3).

**TABLE 3:** Association of gene expression levels with PD adjusted by sex, age and depression.

	OR*	95% CI	*p*
*UBE2K*	1.041	0.758–1.430	0.803
*HSPA8*	0.461	0.278–0.764	0.003
*SKP1*	0.000	0.000–0.000	0.001
*PSMC4*	0.798	0.499–1.278	0.348

OR, Odds Ratio; CI, confidence interval. *Adjusted by sex, age and depression.

### 3.4 Correlation analysis of gene expression

**FIGURE 2:**
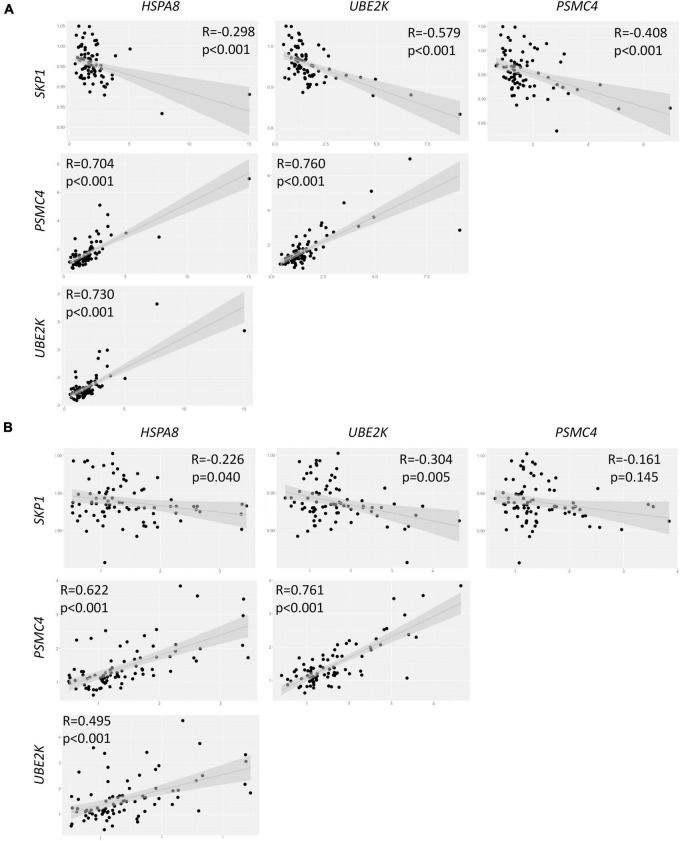
Correlation analysis of gene expression in both control **(A)** and PD **(B)** groups. R = Spearman correlations coefficient.

An exploratory analysis of gene expression within the control group unveiled intriguing interrelationships among the four genes under scrutiny. Specifically, we observed a positive correlation between the expression levels of *HSPA8*, *UBE2K*, and *PSMC4*. Conversely, *SKP1* exhibited a negative correlation with these three genes (Figure 2A). Similar results were observed when testing the PD group, except for the correlation between *SKP1* and *PSMC4*, which was not statistically significant (*p* = 0.165, Figure 2B). Furthermore, a correlation analysis within the PD group revealed no significant correlation between PD severity (measured with UPDRS) and the expression levels of *SKP1A* (*p* = 0.455), *PSMC4* (*p* = 0.655), *UBE2K* (*p* = 0.305) and *HSPA8* (*p* = 0.786).

### 3.5 Predictive utility of gene expression

**FIGURE 3:**
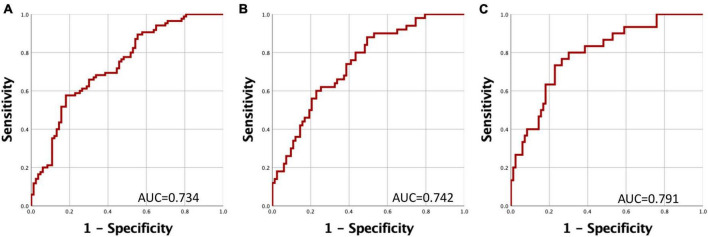
Receiver Operating Characteristic (ROC) Curves for Discriminating Between PD Patients and Controls. The curve represents the relationship between specificity and sensitivity based on the predictive probability derived from gene expression levels of *HSPA8*, *SKP1*, and *PSMC4*. **(A)** Comparison of PD patients versus healthy controls. **(B)** Comparison of PD patients with a disease duration of less than 5 years versus healthy controls. **(C)** Comparison of PD patients with a disease duration of less than 3 years versus healthy controls.

To assess the potential utility of gene expression as a prognostic biomarker for PD, we constructed Receiver Operating Characteristic (ROC) curves based on predictive values derived from logistic regression. We formulated three distinct models, each contingent on the duration of PD evolution (Figure 3). Evaluation of these curves unveiled increasing values for the Area under the Curve (AUC), signifying that a shorter duration of PD evolution positively impacts the predictive value of the model. At a cut-off point of 0.25 it was possible to distinguish between PD individuals and healthy controls with sensitivity and specificity values of 77% and 72.3%, respectively (Figure 3C).

## 4 Discussion

In this study, we delved into the gene expression profiles of *UBE2K*, *HSPA8*, *SKP1*, and *PSMC4* within both control and PD cohorts to evaluate their potential implications in the pathogenesis of PD. Our findings unveiled marked reductions in the gene expression levels of *HSPA8*, *SKP1*, and *PSMC4* in the PD group in comparison to the control group.

The *PSMC4* gene encodes the proteasomal protein S6 ATPase, a constituent of the 19S regulatory subunit essential for the assembly of the 26S proteasome (Dahlmann, 2016). Previous investigations have identified the presence of the *PSMC4* protein in Lewy bodies, demarcating their periphery within dopaminergic neurons of the substantia nigra (Grünblatt et al., 2018). Notably, our observations concerning *PSMC4* gene expression in blood align with prior reports (Molochnikov et al., 2012). Similar reductions in mRNA levels of *PSMC4* within the substantia nigra pars compacta (SNpc) of the brain have been documented in PD patients when compared to controls (Grunblatt et al., 2004; Grünblatt, 2012). The correlation between gene expression levels, protein presence in the SNpc, and localization within Lewy bodies in post-mortem SN samples from PD patients suggests the potential biological relevance of our findings derived from blood samples (Marx et al., 2007; Grünblatt et al., 2018). Recent research indicated a decreased expression of *PSMC4* mRNA, particularly evident after 3 years of disease progression, and established a correlation with disease severity. Nevertheless, this correlation, while noteworthy, did not achieve the requisite strength to be incorporated into a predictive PD classifier model (Rabey et al., 2020).

The *SKP1* gene encodes the SKP1 protein, involved in the formation of the SCF complex, endowed with E3 ubiquitin ligase activity. This intricate assembly plays a pivotal role in identifying target proteins for degradation via the ubiquitin-proteasome system, primarily through interactions with F-box proteins (Zheng et al., 2002). SKP1 exhibits the ability to directly interact with FBXO7, an F-box protein implicated in the regulation of alpha-synuclein (Zhao et al., 2013; Conedera et al., 2016). Perturbations in SKP1 function could potentially contribute to PD development by disrupting the proper degradation of proteins, leading to an accrual of misfolded proteins (Mandel et al., 2012a). Consistent with our findings, reduced expression levels of *SKP1* in both the SNpc and blood have been documented in PD (Grunblatt et al., 2004; Mandel et al., 2009; Molochnikov et al., 2012). It is important to underscore that silencing of *SKP1* has been experimentally demonstrated to promote the accumulation of cytoplasmic inclusions reminiscent of Lewy bodies while concurrently exerting a negative regulatory effect on *HSPA8* gene expression (Fishman-Jacob et al., 2009; Mandel et al., 2012a). *In vitro* studies have further validated that SKP1 deficiency exacerbates PD pathology, culminating in the formation of Lewy body-like inclusions and ensuing neuronal demise (Mandel et al., 2012b). Notably, our identification of a negative correlation between *SKP1* expression and all three genes within our control group implies that SKP1 may potentially exert its effects only in the presence of cellular damage. Consequently, normal *SKP1* expression might not suffice to deter PD progression in the absence of underlying cellular damage.

HSC70 protein, encoded by the *HSPA8* gene, stands as an integral component of Lewy bodies in PD (Grünblatt et al., 2018). This chaperone protein, orchestrates the selective degradation of proteins, maintaining cellular proteostasis through chaperone-mediated autophagy, a process contingent on lysosomes (Nie et al., 2021). Our findings regarding *HSPA8* expression harmonize with prior assessments encompassing transcript and protein levels in both peripheral blood and brain tissues (Grunblatt et al., 2004; Tanaka, 2009; Alvarez-Erviti et al., 2010; Papagiannakis et al., 2015). Nonetheless, it is worth noting that Molochnikov et al. (2012) reported an elevation in *HSPA8* expression levels in PD patients, although the biological significance of this elevation was not explained.

The differential expression profiles observed in our study suggest potential perturbations in protein degradation pathways within our study population, which could underlie the abnormal aggregation of proteins integral to Lewy bodies and consequently impact dopaminergic neuron function.

Our comprehensive ROC curve analysis illuminates the potential utility of *HSPA8*, *PSMC4*, and *SKP1* gene expression levels as effective discriminators between healthy controls and individuals with PD. Similar results in a multi- center study in the German, Italian and Israeli population suggest the ability of a five-gene panel, including *HSPA8*, *PSMC4*, *SKP1* and *UBE2K*, to diagnose early/mild PD in Italy, Germany, and Israel populations (Molochnikov et al., 2012).

As depicted in Figure 3, this discriminatory capacity is further augmented when PD has a shorter duration. These observations collectively indicate that the expression levels of these genes experience significant reductions during the early stages of PD. Importantly, these reductions may be partially ameliorated through medication-induced epigenetic modifications.

Previously, it has been shown that the accuracy of clinical diagnosis for PD could range from 53–74% (Tolosa et al., 2006). More recently, accuracies ranging from 69% to over 90% have been reported in some populations with advanced symptoms (Rizzo et al., 2016). However, a recent study describes an accuracy of 26% in clinical diagnoses for PD in patients with recent symptom onset (Prajjwal et al., 2023). Our model suggests an accuracy of 79.1% for predicting PD in the early stage, as indicated by the AUC values obtained, suggesting it as a promising tool for PD prediction.

One noteworthy limitation of our study resides in the fact that all PD patients were undergoing L-dopa treatment, a factor that has been previously suggested to potentially influence gene expression patterns (Taravini et al., 2016). Reports indicated that L-dopa influenced DNA methylation, resulting in reduced expression of the *SNCA* gene. Nevertheless, it was suggested that its epigenetic influence on other genes could have been possible (Schmitt et al., 2015; Guhathakurta et al., 2017; Song et al., 2017).

The variances in our findings when compared to other populations underscore the necessity of exploring associations between single nucleotide polymorphisms (SNPs) and gene expression to elucidate these disparities (Soldner et al., 2016; Salas-Leal et al., 2021). Consequently, future endeavors should encompass genotyping to modulate transcript variants, shedding light on these discrepancies. Additionally, the execution of cohort studies will be indispensable in delineating the potential utility of our model as a predictive tool for PD.

## Data availability statement

The raw data supporting the conclusions of this article will be made available by the authors, without undue reservation.

## Ethics statement

The studies involving humans were approved by the Comité de ética del Hospital General Dr. Manuel Gea González, Comité de ética del Hospital General 450 and Comité de ética del Hospital General Santiago Ramón y Cajal. The studies were conducted in accordance with the local legislation and institutional requirements. Written informed consent for participation in this study was provided by the participants’ legal guardians/next of kin.

## Author contributions

AS-L: Conceptualization, Methodology, Writing−original draft. SS-P: Data curation, Formal analysis, Methodology, Writing−review and editing. EH-C: Data curation, Formal analysis, Writing−review and editing. LV-V: Investigation, Methodology, Writing−review and editing. EA-S: Investigation, Methodology, Writing−review and editing. FC-J: Investigation, Methodology, Writing−review and editing. EM-H: Data curation, Methodology, Writing−review and editing. OL-L: Data curation, Formal analysis, Writing−review and editing. GQ-C: Data curation, Investigation, Writing−review and editing. OA-C: Data curation, Formal analysis, Writing−review and editing. AS-C: Conceptualization, Formal analysis, Funding acquisition, Supervision, Writing−review and editing. JS-P: Conceptualization, Methodology, Supervision, Visualization, Writing−review and editing.
